# Energy-Efficient and Effective MCF-7 Cell Ablation and Electrothermal Therapy Enabled by M13–WS_2_–PEG Nanostructures

**DOI:** 10.3390/ma17184624

**Published:** 2024-09-20

**Authors:** Maria P. Meivita, Fitya S. Mozar, Shao-Xiang Go, Lunna Li, Natasa Bajalovic, Desmond K. Loke

**Affiliations:** 1 Department of Science, Mathematics and Technology, Singapore University of Technology and Design, Singapore 487372, Singapore; 2 Thomas Young Centre and Department of Chemical Engineering, University College London, London WC1E 7JE, UK

**Keywords:** cancer therapy, thermal ablation, 2D materials, WS_2_, PEG, MCF-7, M13 phage

## Abstract

Thermal agents (TAs) have exhibited promise in clinical tests when utilized in cancer thermal therapy (TT). While rapid degradation of TAs may address safety concerns, it limits the thermal stability required for effective treatment. TAs, which possess exceptional thermal stability, experience gradual deterioration. There are few approaches that effectively address the trade-off between improving thermal stability and simultaneously boosting material deterioration. Here, we control the thermal character of tungsten disulfide (WS_2_)-based 2D materials by utilizing an M13 phage through Joule heating (the M13–WS_2_–PEG nanostructures were generated and termed a tripartite (T) nanostructure), and developed a T nanostructure-driven TT platform (we called it T-TT) for efficient thermal ablation of clinically relevant MCF-7 cells. A relative cell viability of ~59% was achieved, as well as onset time of degradation of ~0.5 week. The T-TT platform also discloses an energy density of 5.9 J/mL. Furthermore, the phage-conjugated WS_2_ can be utilized to achieve ultrasound imaging for disease monitoring. Therefore, this research not only presents a thermal agent that overcomes TA limitations, but also demonstrates a practical application of WS_2_-type material system in ultra-energy efficient and effective cancer therapy.

## 1. Introduction

Cancer thermal therapy (TT) employs thermal agents (TAs) to induce local hyperpyrexia, effectively eliminating tumors. This minimally invasive and highly effective therapeutic approach has demonstrated significant effectiveness in both preclinical and clinal investigations [1,2,3,4]. The International Agency for Research on Cancer (IRAC) 2022 data indicates that cancer is a growing issue, particularly affecting disadvantage communities, underscoring the urgent need to address global disparities in cancer treatment and outcomes. In 2022, 20 million new cancer cases and 9.7 million deaths were reported, with 53.5 million survivors within 5 years. Cancer affects 20% of individuals, with 11% of men and 8% of women succumbing to the disease [5]. In a recent clinical study, the TT-based tumor ablation procedure was effective in numerous patients, with no negative impact on organ functions, significant side effects, or severe repercussions [6,7]. This therapy strategy is expected to have a significant, if not groundbreaking, impact on clinical outcomes, supported by these promising results, as well as the clinical endorsement of metal oxide nanoparticles for advanced TT in Europe [8,9,10]. Nevertheless, the usage of traditional metal nanoparticles has a drawback in terms of potential toxicity and biocompatibility due to the presence of metal nanoparticles [11,12]. However, it is challenging to create traditional TAs that possess both rapid degradability, meaning they degrade quickly to address safety concerns, and exceptional thermal effects, i.e., high thermal conversion efficiency and/or thermal stability, to achieve beneficial therapeutic outcomes [13,14,15]. 

Currently, nanoagents with a high thermal stability upon the application of input stimuli, e.g., inorganic nanomaterials, are a type of TAs used for preclinical investigations [16,17,18]. Moreover, experiments have demonstrated the antitumor effectiveness of TAs with chemotherapeutic drugs, e.g., diamminedichloroplatinum, paclitaxel, and interleukin-12 [19,20,21,22]. The attainment of an excellent antitumor effect was exhibited in these experiments. However, traditional high-thermal-stability TAs generate excretion concerns and biosafety problems, since the TA disintegrates with difficulty or slowly [23,24,25]. Another type of TA that degrades quickly, e.g., the clinically approved indocyanine green, causes fewer concerns regarding biocompatibility and safety [26,27,28]. Nevertheless, the rapid degradation of traditional quick-degradable TAs undermines their thermal stability, which is necessary for enhanced therapeutic effectiveness [29,30]. The thermal function would typically dissipate after a few seconds or following repeated stimulation. Moreover, the TA deterioration is aggravated by the traditional input stimulus [31,32]. Clinical complications such as a high liver and renal load occur, although an increased TA dose results in a stronger TT. Thus, a substantial hurdle to realizing the therapeutic potential of TT is described by the contradiction between increasing the thermal stability, and simultaneously, enhancing the material degradation of conventional TAs [33,34].

Tungsten disulphide (WS_2_) has been extensively included into functional TAs with diverse nanostructures due to its biocompatibility and biodegradability as a two-dimensional (2D) material [35,36,37]. The WS_2_ can be readily oxidized and decomposed to benign components, which increases the TA safety [38]. As a result, traditional WS_2_ types are one of the TAs with quick degradation but poor thermal stability. Despite numerous modifications aimed at enhancing the stability and thermal efficiency of the traditional WS_2_ type [39,40], these advantages are accompanied by prolonged degradation. In addition, the cellular uptake of 2D materials, e.g., MoS_2_ and black phosphorus (BP), has been demonstrated in recent studies [41,42,43,44,45]. The recent works disclosed the successful integration of 2D materials in cancer cells. The M13 is a cylindrical, ~880 nm long, and 5–6 nm in diameter bacteriophage composed of specified proteins [46,47,48,49]. Based on the fact that the M13 phage is effective at targeting cancer cells and that polyethylene glycol (PEG) can enhance the stability and biocompatibility of WS_2_, we propose that combining WS_2_ with M13-conjugated PEG could enhance the thermal performance of M13–WS_2_–PEG nanostructures (referred to as tripartite (T) nanostructures), while still allowing for rapid degradation.

**Figure 1:**
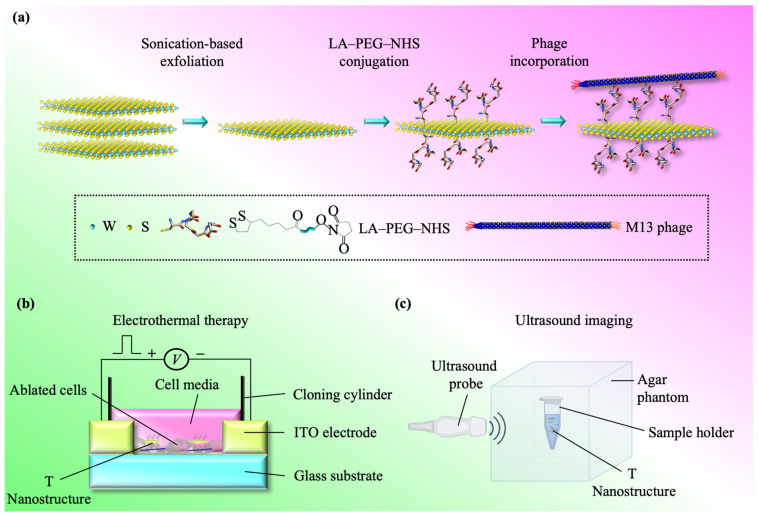
A thermal therapeutic platform driven by T nanostructures. (**a**) Schematic diagram of the approach used for the fabrication of the WS_2_-conjugated PEG and the subsequent phage integration. The illustration of the M13 was adapted from the reference [50]. (**b**,**c**) Schematic illustrations of the T nanostructure utilized for (**b**) therapeutic efficacy studies, as well as (**c**) ultrasound imaging.

Herein, we show that by manipulating Joule heating, we can regulate the thermal characteristics of WS_2_ using a phage-based approach. Additionally, we develop a T nanostructure-type TT (T-TT) platform that effectively destroys MCF-7 cells (Figure 1). The approach is based on the idea of utilizing the phage to induce the assembly of a large amount of conductive WS_2_ on cancer cells through a high specificity of binding of the WS_2_ nanosheet to the cancer cell, which enables strong Joule heating of the T-TT platform upon the application of alternating current (AC) stimuli and, concomitantly, retaining quick degradation. The T-TT exhibits a relative cell viability of ~59%, along with an onset time of degradation of ~0.5 weeks. An energy density of 5.9 J/mL was attained. We show that by using the phage-conjugated WS_2_, the TA may be utilized as a resilient, adaptable, and benign imaging tool to assess treatment results. We have collectively proposed a technique that is both energy-efficient and successful in resolving the contradiction between raising the thermal stability and enhancing the material degradation of TAs.

## 2. Materials and Methods

*Cell culture*. MCF-7 human breast cancer cells were cultured in Dulbecco’s Modified Eagle’s Medium (DMEM) with L-glutamine (Nacalai Tesque, Nakagyo-ku, Kyoto, Japan) and 7% Fetal Bovine Serum (FBS) (Gibco, Thermo Fisher Scientific, Waltham, MA, USA) at 37 °C in a humidified incubator with 5% CO_2_. MCF-10A non-tumorigenic breast epithelial cells were cultured in DMEM/F12 medium supplemented with 10% FBS and 20 ng/mL of epidermal growth factor (Gibco). Additionally, 500 ng/mL hydrocortisone and 10 μg/mL insulin (Sigma Aldrich, St. Louis, MO, USA) were added to the medium. The MCF-10A cells were also maintained in the incubator under the same conditions.

*Escherichia coli (E. coli) and M13 bacteriophage propagation*. Escherichia coli (*E. coli*, 5-alpha F’Iq competent) was purchased from New England Biolabs (NEB, Ipswich, MA, USA) to propagate M13 bacteriophage. An overnight culture (O.C.) of *E. coli* was prepared using tetracycline (TET) and grown for 4–6 h at 37 °C with 90 rpm shaking. The O.C. was then used to inoculate a Lennox broth (LB) medium (Sigma Aldrich, St. Louis, MO, USA). The culture was incubated until the optical density at 600 nm (OD_600_) reached 0.4. M13 bacteriophage (ATCC 15669-B1) was revived following ATCC protocols and allowed to grow overnight. Amplifications were performed for the first and second precipitations according to the ATCC protocol. Finally, the concentration of the M13 bacteriophage was quantified using a μDrop plate (Thermo Fisher Scientific, Waltham, MA, USA).

*T nanostructure preparation*. The tungsten disulfide, WS_2_, in deionized (DI) water with a material concentration of 25 µg mL^–1^ was utilized (2D Semiconductors, Scottsdale, AZ, USA). Additionally, 1.87 mL of WS_2_ aqueous solution was sonicated for 30 min before the T nanostructure conjugation. For the PEG coating, the Lipoic acid–polyethylene glycol–N-hydroxysuccinimide (LA–PEG–NHS) solution with a polymer concentration of 10 mg mL^–1^ was used (Nanocs Inc., Boston, MA, USA). Thereafter, 10 µL of LA–PEG–NHS solution was added to the sonicated WS_2_ solution. The mixture was then placed on a shaker at 25 °C for 48 h. The sterile M13 bacteriophage solution with a phage concentration of 10^12^ pfu mL^–1^ was harnessed. Thereafter, 112 µL of M13 solution was added to the mixture, incubated, and shaken at 25 °C for an additional 48 h. Finally, the conjugated T nanostructure mixture or solution was washed three times through the centrifugation process at 10,000 rpm to achieve the T nanostructures.

For each stability or ablation experiment, 0.1 mL of T nanostructure solution was resuspended in 0.9 mL of Dulbecco’s Phosphate-Buffered Saline (DPBS) solution to generate the T nanostructure-DPBS sample with 10 vol% T nanostructure solution. To create the T nanostructure-DPBS sample with 30 vol% T nanostructure solution, 0.3 mL of T nanostructure solution was resuspended in 0.7 mL of DPBS solution, for each stability or ablation experiment.

*Material characterization*. The atomic force microscopy (AFM, Bruker Dimension Icon system, Billerica, MA, USA) was utilized to investigate the morphology of the WS_2_ nanosheets and T nanostructures. The samples were drop-casted onto a silicon, Si, substrate and examined using a scan size of 1.5 μm × 1.5 μm. Gwyddion software version 2.6.2. was used to measure the sample thickness. The tapping operational mode and the Bruker OTESPA-R3 probe were utilized for AFM. Additionally, the T nanostructure was imaged using the transmission electron microscopy (TEM, FEI Talos F200 system, Hillsboro, OR, USA), and with an accelerating voltage of 120 kV. For the TEM preparation, the negative staining was applied, and the samples were drop casted on a carbon-coated Cu grid. In the stability experiment, T nanostructure-DPBS samples with 10 and 30 vol% T nanostructure solution were utilized, and maintained at 37 °C in a humidified incubator with 5% CO_2_. Absorbance measurements of the T nanostructure were taken at λ = 500–600 nm at specific time intervals. For the thermal analysis, hotspot locations were identified using a lock-in infrared (IR) thermography with the ELITE system (Thermo Fisher Scientific, Waltham, MA, USA), while a direct-current (DC) electrical stimulus was applied to a bulk WS_2_.

*Cell cytotoxicity studies*. MCF-7 and MCF-10A cells were seeded in 96-well plates at a cell density of 3 × 10^3^ cells per well and incubated at 37 °C for 24 h. The medium was then aspirated. Cells were treated with various T nanostructure concentrations, while cells only served as the experimental control. The cells were further incubated at 37 °C for an additional 24 or 48 h. To assess the cytotoxicity, the WST-1 staining assay was utilized. After the incubation, the materials were removed from each well, and the cells were washed twice with the DPBS. Subsequently, 100 μL of the WST-1 staining assay was added to each well and allowed to incubate for 2 h. Finally, the optical density of cell samples was measured at λ = 450 nm using a microplate absorbance reader (Thermo Scientific Multiskan GO, Waltham, MA, USA).

*Electrothermal experiment*. MCF-7 and MCF-10A cells were seeded onto the indium tin oxide (ITO) subsystems, which comprised left and right 650 nm-thick ITO electrodes grown on a glass substrate (Latech, Singapore). The glass substrate with the ITO electrode was then mounted with a cloning cylinder using a silicone adhesive (Sigma Aldrich, St. Louis, MO, USA). Each subsystem was plated with MCF-7 and MCF-10A cells at a density of 3 × 10^3^ per well and incubated for 24 h. Subsequently, T nanostructure-DPBS samples with 10 and 30 vol% T nanostructure solution were added into subsystems, while the subsystem with cells only was used as the experimental control. After 24 h, electrical stimuli with input amplitudes of 1.0, 2.0, and 5.0 V as well as stimulus length of 2 μs; comprising 1000 input bursts were applied to the T-TT system. To prevent medium depletion, 50 μL of DMEM was added to each well. Following the application of stimuli, the WST-1 assay was utilized after 24 h to measure the cell viability.

*Ultrasound (US) imaging*. For US imaging, an agar phantom was created by inserting a 2-mL Eppendorf tube in the agar. Thereafter, 10 g of the agar was dissolved in 250 mL of water and heated at 100 °C with stirring. The resultant hot solution was added to a square plastic mold, and the Eppendorf tube was positioned near the center of the agar to create a hollow channel. Subsequently, Eppendorf tubes containing 2 mL of pristine DMEM samples and 2 mL of T nanostructure-DMEM samples with 30 vol% T nanostructure solution were inserted into the channel. Finally, ultrasound images were obtained using an ultrasound scanner (Ultrasonix SonixTouch, BC, Canada) equipped with a transducer (Ultrasonix PA7–4/12 probe, BC, Canada).

## 3. Results

*Synthesis and characterization of T nanostructures*. We investigate how changes in material type from pristine WS_2_ nanosheets to WS_2_ nanosheets with PEG and M13 phage are linked to changes in material characteristics. WS_2_ nanosheets are generated by the ultrasonication-assisted liquid exfoliation of a bulk WS_2_.

Selecting the suitable solvent in liquid exfoliation can greatly enhance both the synthesis efficacy and production quantity of nanomaterials. Solvents, e.g., N-methylpyrrolidone (NMP) are effective at exfoliating nanomaterials, viz., transitional-metal dichalcogenides (TMDCs) [51]. On the other hand, solvents, i.e., isopropyl alcohol (IPA), generate nanosheets with specific thicknesses, although these solvents are generally less effective for exfoliating bulk TMDCs since the solvent exhibits long sonication times [52]. By choosing suitable solvents, dispersibility improves, increasing production quantity. For example, the effective solvent, i.e., NMP, achieves 0.3 mg/mL material concentration for MoS_2_, while IPA attains 0.06 mg/mL material concentration for BN [53].

**Figure 2:**
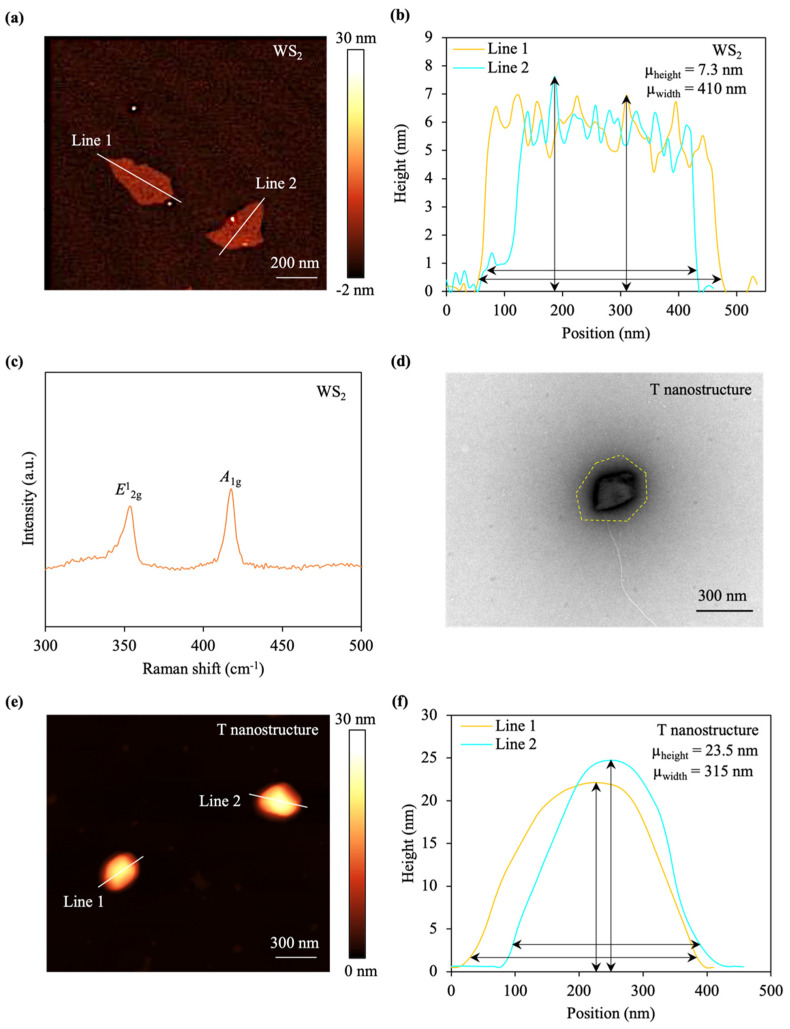
T-based nanostructure characterization. (**a**) Atomic force microscopy (AFM) image of WS_2_ nanosheets. (**b**) Height profiles of the WS_2_ nanosheet along the white lines in (**a**). The WS_2_ nanosheets exhibit a ±9.5% variation from the average values in the thickness and lateral dimension. (**c**) Raman spectra of the bulk WS_2_. (**d**) Transmission electron microscopy (TEM) image of the T nanostructure. The yellow dashed area consists of the head or sheet-like structure. (**e**) AFM image of the T nanostructure. (**f**) Height profiles of the T nanostructure, along the white lines in (**e**). The T nanostructures demonstrate a ±9.0% variation from the mean values in both thickness and lateral dimension.

The WS_2_ nanosheets show an average lateral size of ~410 nm (Figure 2a, Supporting Figure S1) and an average thickness of ~7.3 nm (Figure 2b), indicating a stack of two triple layers of WS_2_, as disclosed by the atomic force microscopy (AFM) image, sectional plot, and transmission electron microscopy (TEM) image.

The Raman spectra of the bulk WS_2_ were measured to examine the crystal quality and structure of the WS_2_. Two characteristic Raman peaks, viz., the *E*^1^_2g_ at 350 cm^–1^ and the *A*_1g_ at 420 cm^–1^, were discerned in the Raman spectra of the WS_2_ (Figure 2c). The experimentally distinguished Raman peak at 350 cm^–1^ results from the degenerate mode of the *E*^1^_2g_ symmetry, which conforms to in-plane transversal and longitudinal vibrations of the sublayer in opposite directions. The third mode opposite-in-phase out-of-plane vibrations of the sublayer of the *A*_1g_ symmetry lead to the peak at 420 cm^–1^ [54,55,56].

The biocompatibility and stability of the WS_2_ nanosheet were enhanced through the utilization of PEG [57]. Furthermore, in order to enhance the ability of WS_2_ to target cancer cells, a bacteriophage M13, which selectively assembles on the HER2, was linked to the PEG molecule [58,59]. The HER2 is an appealing therapeutic target because it belongs to the integrin family and is unregulated in both angiogenic endothelial cells and tumor cells [60,61]. The M13 phage was conjugated to the PEG molecule via an amine reaction, whereas the WS_2_ nanosheet was tethered to PEG molecules through disulphide binding. The conjugation was achieved by employing an LA–PEG–NHS combination as a linker connecting the M13 and PEG molecule. The LA molecule was attached to the WS_2_ nanosheet through a disulphide binding [62], whereas the NHS group of the LA–PEG–NHS interacted with the amide group on the M13 phage [63]. Finally, as shown in Figure 1a, the M13–WS_2_–PEG nanostructure was created and termed T nanostructure.

To examine the grafting of the LA–PEG–NHS on the WS_2_ or M13 surfaces, Fourier transform infrared (FTIR) spectroscopy was harnessed (Supporting Figure S2). The FTIR spectra of the T nanostructure solution showed the characteristic stretching vibration of the carbonyl group in PEG at approximately 1090 cm^–1^, suggesting the presence of PEG on the WS_2_ surface [64,65]. Another peak at ∼1640 cm^−1^ corresponds to a carbonyl stretch (C=O) associated with amide bonds, which indicates a PEG–M13 phage reaction [66]. Lastly, the peak at ∼530 cm^−1^ is characteristic of WS_2_ [67].

Spectroscopic measurements, such as FTIR, enable the assessment of biochemical changes in cellular components at the single-cell level with nanomaterials for different cell lines. In a work on three human breast cell lines, i.e., MCF-10A, MCF-7, and MDA-MB-231, treated with nanomaterials, FTIR analysis revealed distinct bands indicating changes in RNA/DNA, lipids, and amide I and II vibrations [68]. The most significant effect was observed in the MCF-7 cell line, where bands corresponding to DNA/RNA vibrations exhibited the highest intensity.

Furthermore, FTIR spectroscopy can differentiate cancer cells with and without nanomaterials. In the investigation, three cell lines were examined, and each cell line was treated with varying material concentrations [68]. The FTIR spectra of these cell lines with and without nanomaterials displayed similar peaks. Minimal changes in relative band intensity, width, and position in the spectra were recorded under different conditions in the cell lines with nanomaterials compared to those of controls, i.e., cell lines without nanomaterials, after 24 h incubation. However, a detailed analysis of the spectra collected for the three cell types with nanomaterials incubated for 48 h under different experimental conditions revealed more significant changes than that of the control, especially at higher material concentrations.

The T nanostructure demonstrates a flower-type morphology and a head-based material sheet, as revealed by the TEM image (Figure 2d). The average lateral size of the T nanostructure is approximately 315 nm (Figure 2e), which is smaller than that of the pristine WS_2_ nanosheet, as a result of the sonication process that could partially break down nanosheets. The sectional plot elucidates that the T nanostructure thickness is ~23.5 nm (Figure 2f), thicker compared to that of the pure WS_2_ nanosheet, owing to the existence of the M13 phage structure and the PEG coating. Experiments have revealed that the M13 shows a diameter of ~6 nm, while a thickness of ~10 nm is exhibited by the PEG [69,70].

**Figure 3:**
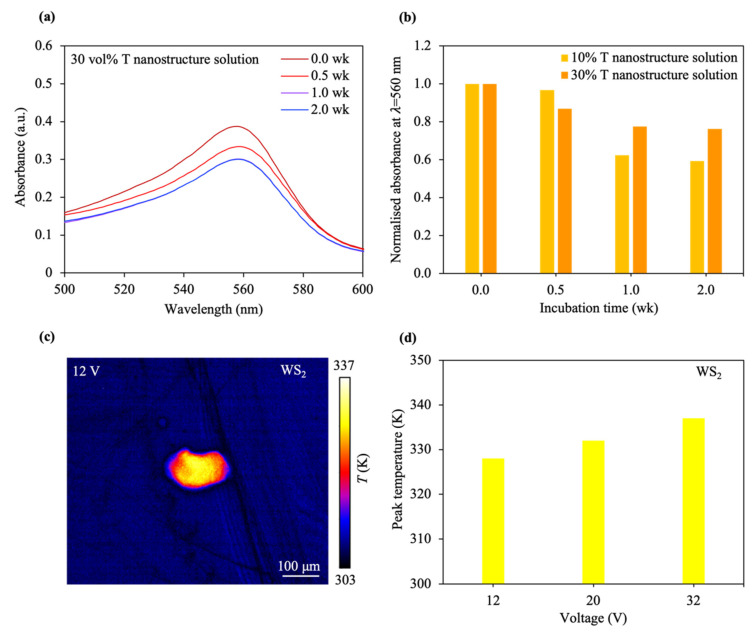
Stability and thermal-based effects of T nanostructures. (**a**) UV-vis absorbance spectra of the T nanostructure-DMEM sample with 30 vol% T nanostructure solution and for different weeks. (**b**) The normalized absorbance at λ = 560 nm was measured for T nanostructure-DMEM samples containing 10 and 30 vol% T nanostructure solutions at various time intervals. (**c**) Thermographic map of a bulk WS_2_ after the application of a 12 V-strong input stimulus. (**d**) Analysis of the peak temperature fluctuation in the bulk WS_2_ sample under varied stimulus amplitudes.

*T nanostructure degradation time and thermal phenomena*. Encouraged by the material signature data, we investigate the degradation characteristics of T nanostructures (Figure 3a). The TA can be grouped into weak-to-moderate degradation-dominated TAs and strong degradation-dominated TAs, depending on the absorbance signature. In a weak-to-moderate degradation-dominated TA, an intermediate-to-large output absorbance occurs, e.g., a normalized absorbance above 0.70 is achieved, while a small output absorbance results, viz., the attainment of a normalized absorbance below 0.70, in the case of a strong degradation-dominated TA. An ultraviolet visible-near infrared (UV–Vis–NIR) spectrometer was used to measure the output absorbance of material samples. The T nanostructure solution was added to the DMEM solvent at week 0 and the output absorbance of the material sample was read at different weeks. The normalized absorbance variation at different times for material samples with different T nanostructure solution concentrations is shown in Figure 3b. After a 1 week-long material incubation in the cell media, and when the material sample with a low-concentration T nanostructure solution is utilized, the normalized absorbance becomes small, e.g., ~0.60, resulting in the occurrence of a strong degradation-dominated TA. In contrast, a moderate degradation-dominated TA appeared for the case after a 1 week-long material incubation in the cell media, and when the material sample with a high-concentration T nanostructure solution was harnessed. The normalized absorbance obtained was intermediate, viz., 0.75. The thermal distribution of the bulk WS_2_ created by a direct-current (DC) stimulus and the peak temperature variation for different input amplitudes were examined, as shown in Figure 3c,d. When a stronger stimulus is administered, an increased peak temperature arises. Moreover, the peak temperature increases with an increase in the stimulus amplitude and WS_2_ thickness for the T nanostructure-with-cancer-cell model (Supporting Figure S3, Table S1), which means that the thermal signature of the WS_2_ can be modulated.

**Figure 4:**
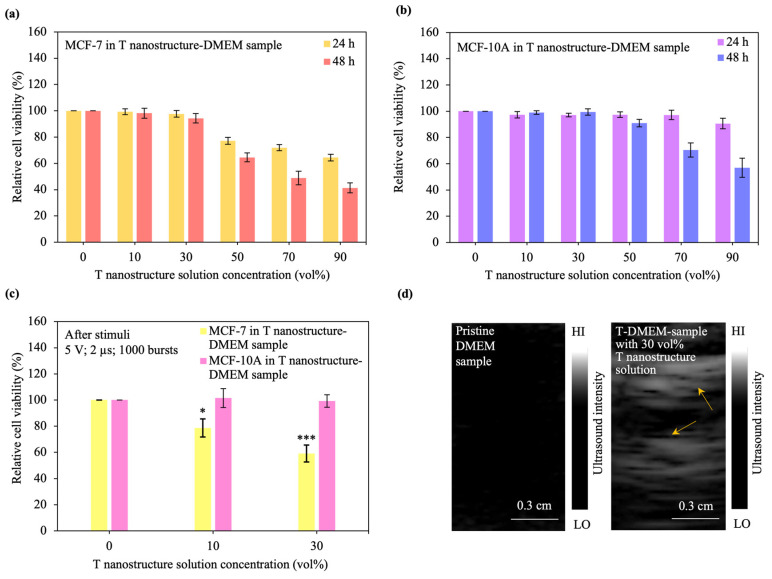
T-based cancer cell ablation and ultrasound imaging. (**a**,**b**) Variation of relative viabilities of (**a**) MCF-7 and (**b**) MCF-10A cells in T nanostructure-DMEM samples with different T nanostructure solution concentrations incubated for 24 and 48 h. The statistical significance of the relative cell viabilities can be determined by referring to Supporting Table S1. (**c**) Relative viability variations for the MCF-7 and MCF-10A cells in T nanostructure-DMEM samples with different T nanostructure solution concentrations after AC input stimuli. The significance values were computed using the Student’s t-test for *n* = 6 and are denoted as follows: * (*p* < 0.05) and *** (*p* < 0.001). The non-significance values were unmarked. The error bars represent the standard error of the mean (SEM) where *n* = 6. (**d**) Ultrasound imaging of the pristine DMEM sample and T nanostructure-DMEM sample with 30 vol% T nanostructure solution. HI denotes high ultrasound intensity, while low ultrasound intensity is represented by LO.

*Cancer cell ablation driven by T nanostructures*. The degradation and thermal character results provided encouragement to examine the impact of the T nanostructure on cytotoxicity. The cytotoxicity of WS_2_ nanosheets is associated with surface phenomena [71,72]. We conceive that the cytotoxicity of T nanostructures can be controlled by the material concentration and cell sample as they modulate the WS_2_ surface process. In order to evaluate this idea, we establish the level of destructiveness of the nanostructure by examining the cell sample. Consequently, replacing the cell sample from MCF-7 cells with T nanostructures and from MCF-10A cells with T nanostructures results in a reduced level of destructiveness. Our focus is on investigating how nanostructures affect cell viability; therefore, we measure changes in cell viability after adding the materials. Figure 4a demonstrates that cell viability is dependent on both the level of destructiveness and the material concentration. A high level of destructiveness is found to be the least suitable, while a low level of destructiveness is the most suitable. When MCF-7 cells are treated with T nanostructures, the cell viability stays high as long as the material concentration is below 50%. Nanostructures that cause minimal damage are capable of maintaining a low level of cytotoxicity, leading to a non-toxic nanostructure. However, as the material concentration grows, there is a noticeable decrease in cell viability due to an increase in the level of destructiveness. This implies that the nanostructure possessing a significant level of destructiveness has the potential to arise and exhibit cytotoxic effects.

Figure 4a,b and Supporting Table S2 demonstrate that cell viability varies for different degrees of destructivity and cell samples. In this case, experiments with MCF-10A cells incubated for 24 h revealed that large material concentrations may be used to attain high cell viability. Conversely, when the T nanostructure is injected to MCF-7 cells incubated after 24 h, the surface chemistry between the cancer cell and the WS_2_ nanosheet can reach a level that induces cell death. However, it is possible that the material concentration utilized is large enough to cause cell death for a high cell population, leading to low cell viability.

Experiments have shown that cancer cells exposed to traditional nanomaterials exhibited physical damage [73,74,75]. Scanning electron microscopy (SEM) was utilized to analyze the cellular morphology changes in MCF-7, liver cancer (HepG2), and cervical cancer (CaSki) cells with nanowires [73]. The SEM images revealed that these cells with conventional nanowires displayed mechanical damage caused by cell-structure disruption induced by the nanostructure, as well as significant changes in cell morphology.

The cell viability upon the application of AC stimuli was examined and the results were shown in Figure 4c. Experiments used in this research have demonstrated that cell viability remains high when the T nanostructure solution concentration of T nanostructure-DMEM samples is below 50 vol%. To achieve high electrical conductance for strong Joule heating and high cell viability, we choose to utilize cells in T nanostructure-DMEM samples with 10 and 30 vol% T nanostructure solutions. When AC stimuli are applied to MCF-7 cells in T nanostructure-DMEM samples, the relative cell viability decreases. In contrast, when AC input stimuli are administered to MCF-10A cells in T nanostructure-DMEM samples, which means that the nanostructure has a low level of destructiveness, nanostructures can be considered as non-destructive, and no substantial variation in the extent of cancer cell killing occurs. Moreover, to attain a higher extent of cancer cell destruction and stronger thermal performance, a higher level of destructiveness should result from an increasing stimulus amplitude (Supporting Figure S4).

## 4. Discussion

Encouraged by the excellent cancer cell-targeting ability of T nanostructures, we investigate further the material signature of the T nanostructure by ultrasound imaging. Experiments have demonstrated that a stronger ultrasound signal results when the sample is altered from a pure phantom or tissue block to a phantom or tissue block with nanostructures owing to a rattle-type process [76,77,78]. The imaging setup is shown in Figure 1c. To examine the material signature, we utilized an ultrasound probe to record ultrasound images and variations in the ultrasound signal (Figure 4d). When the pure phantom block was used, the ultrasound signal became weak. Conversely, the ultrasound signal became strong for the case when the phantom block with T nanostructures was utilized, indicating a structural rattle-type process. These results suggest that the T nanostructure is a multifaceted tool used for monitoring disease progression, which can substantially enhance clinical-treatment planning.

The nanosized TA design is difficult for applications such as the TT owing to various requirements: (i) low cell viability, (ii) short onset time of degradation, and (iii) low energy density. For now, none of the current TAs fulfill all of the necessary requirements. According to the examples shown in this research, the current condition of the T nanostructure seems to fulfill most of these requirements with a low cell viability. The main enhancement in the T nanostructure that enables these applications is the achievement of a relative cell viability of 59% in the T-TT platform (these results are obtained from MCF-7 cells in the T nanostructure-DMEM sample with 30 vol% T nanostructure solution and with 5.0 V-strong input stimuli), which is ~28.5 percentage points lower than the average of 87.5% for currently available electrothermal-based therapeutic systems (Supporting Figure S5, Table S3). Consequently, a significant population of cancer cells are eradicated, resulting in the successful implementation of a cancer treatment. Moreover, an onset time of degradation of 0.5 weeks in the T nanostructure was attained, which was ~50% lower than the average of 1.0 weeks for state-of-the-art nanosized TAs (Supporting Figure S6, Table S4). This result allows a quick material discharge for attaining safe cancer therapy. Furthermore, the T-TT platform demonstrates a low energy density of 5.9 J/mL (the equation, energy density = number of pulses (*n*) × electric field (*V*/*d*) × current density (*J*) × pulse width (*t*) = 1000 × 500 V/cm × 0.59 A/cm^2^ × 2 μs, was used; electric field = input voltage (*V*)/electrode distance (*d*) = 5 V/0.1 mm; current density = current (*I*)/cloning cylinder area (*A*) = 0.1 A/0.17 cm^2^), which is ~83.14% lower than the average of 35.0 J/mL for current electrothermal-variety therapeutic systems (Supporting Figure S7, Table S5). The excellent performance enables therapeutic methodologies to utilize low input energy for achieving highly energy-efficient cancer therapy.

Advanced microscopic techniques offer powerful methodologies for analyzing and identifying cellular membrane nanomechanical properties, thereby enabling effective cancer treatment. Techniques such as atomic force microscopy (AFM) and optical tweezers have generated accurate data to help reveal how soft matter or cellular systems respond to external forces [79]. These methods allow for a detailed examination of cell plasticity and membrane nanomechanical properties by applying controlled forces to cancer cells.

Cell ablation techniques, viz., those utilizing nanomaterials, have leveraged nanomechanical properties to destroy cancer cells. For example, carbon nanotubes enhance cancer cell thermal ablation by creating a “lightning rod” effect that amplifies electric fields [80]. This phenomenon creates pores in cancer cell membranes even at relatively low electric-field strengths, which facilitates therapeutic agent delivery and aids in cancer cell destruction.

## 5. Conclusions

This work focuses on creating a thermal agent with both fast degradation and high thermal performance for robust thermal-based therapy. Thermal agents with high thermal performance often face safety concerns during clinical translation, while those that degrade quickly have exhibited promising clinical applicability. To address this issue, researchers in this work have developed a technique that simultaneously displays high thermal performance and maintains fast degradation using WS_2_-based thermal agents, which has the ability to induce strong Joule heating. The T nanostructures, generated by integrating PEG and WS_2_ with M13 phage, showed improved thermal performance, and at the same time, maintained rapid degradation. This approach addresses the thermal agent dilemma by incorporating WS_2_ with PEG and allowing for enhanced material stability. The addition of M13 phage enhances the cell targeting ability of T nanostructures for thermal-based therapy. The T nanostructures can be utilized in ultrasound imaging for disease monitoring. This approach highlights the clinical potential of WS_2_-type materials in ultrasound imaging-based, thermal therapy-enhanced combination cancer therapy. The T nanostructure can be customized with different components and strategically integrated with various therapeutic modalities for successful combinatorial cancer treatment.

These low relative cell viability, short onset time of degradation, along with low energy density are attained through a phage-based scheme that modulates the Joule heating of the WS_2_. An alternative approach that has the potential to effectively address the tradeoff between improving thermal stability and increasing degradability is the usage of T nanostructures. Moreover, this research establishes the possibility of using WS_2_ in cancer treatment and ultrasound imaging, taking into account the remarkable capabilities of TT in medical practice and the significant influence of WS_2_ in biomedical fields.

## Data Availability

The authors declare that data supporting the finding of this study are available within the article and the Supporting Information. Other data are available from the corresponding authors upon reasonable request.

## Supplementary Materials

The following supporting information can be downloaded at: https://www.mdpi.com/article/10.3390/ma17184624/s1, Table S1: Thermoelectric properties of the cell-layer/T-nanostructure model utilized in electrothermal simulations; Table S2: Statistical significance analysis of the MCF-7 and MCF-10A cell cytotoxicity for T nanostructure-DMEM samples with different T-nanostructure solution concentrations (10–90 vol%) compared to the control (cells only); Table S3: References for Figure S5; Table S4: References for Figure S6; Table S5: References for Figure S7. Figure S1: Transmission electron microscopy (TEM) images of the WS_2_ nanosheet and M13 phage; Figure S2: Fourier-transform infrared (FTIR) spectrum of the T nanostructure sample; Figure S3: (a) Thermal-distribution analysis snapshot of the cell-layer/T-nanostructure model. (b) Variation of the peak temperature in the cell layer observed for different stimulus amplitudes. (c) Variation of the peak temperature in the cell layer observed as a function of WS_2_ thickness for 5 V input stimulus; Figure S4: Relative viabilities of MCF-7 cells in T nanostructure-DMEM samples for different stimulus amplitudes; Figure S5: Comparison of the relative cell viability between the T-TT platform and existing electrothermal-based therapeutic systems; Figure S6: Comparison of the degradation time of the T nanostructure with state-of-the-art thermal agents in a physiological media; Figure S7: Comparison of the energy density between the T-TT platform and existing electrothermal-based therapeutic systems.
