# Supplementary material for: Energy-Efficient and Effective MCF-7 Cell Ablation and Electrothermal Therapy Enabled by M13–WS2–PEG Nanostructures
Source: Materials (Basel). 2024 Sep 20;17(18):4624. doi: 10.3390/ma17184624 (PMC11433225; doi:10.3390/ma17184624)
Supplement: Supplementary file 1 [file materials-17-04624-s001.zip › materials-3139242-supplementary.pdf]

Supporting Information

Energy-efficient and effective MCF-7 cell ablation and electrothermal therapy enabled by M13–WS_2–PEG nanostructures

Maria P. Meivita,[1] Fitya S. Mozar,[1] Shao-Xiang Go,[1] Lunna Li,[2] Natasa Bajalovic,[1] Desmond K. Loke[1]

[1]*Department of Science, Mathematics and Technology, Singapore University of Technology and Design, Singapore 487372, Singapore*

[2]*Thomas Young Centre and Department of Chemical Engineering, University College London, London WC1E 7JE, United Kingdom*

[*]*Correspondence and requests for material should be addressed to N.B. (e-mail: natasa_bajalovic@sutd.edu.sg) or D.K.L (e-mail: desmond_loke@sutd.edu.sg).*

Table S1. Thermoelectric properties of the cell-layer/T-nanostructure model utilized in electrothermal simulations.

Material	Isotropic thermal conductivity (W/mK)	Isotropic resistivity (Ω cm)
**SiO_2**	1.4	10^{16}
**ITO**	4	0.0001
**M13/PEG**	0.2	0.1
**Cell in DMEM**	0.63	97.66
**WS_2**	140	1.52

Table S2. Statistical significance analysis of the MCF-7 and MCF-10A cell cytotoxicity for T nanostructure-DMEM samples with different T-nanostructure solution concentrations (10 – 90 vol%) compared to the control (cells only). Significance was fixed based on the Student's t-test and indicated as: * ($p < 0.05$), ** ($p < 0.01$), *** ($p < 0.001$), **** ($p < 0.0001$). Non-significant results were unmarked.

**MCF-7**		**Concentrations (vol%)**				
		**10**	**30**	**50**	**70**	**90**
**24 h**	relative to control			***	****	****
**48 h**	relative to control			***	****	****

**MCF-10A**		**Concentrations (vol%)**				
		**10**	**30**	**50**	**70**	**90**
**24 h**	relative to control					
**48 h**	relative to control			*	**	**

Table S3. References for Figure S5.

Ref No.	Reference	Electric field (V/cm)
1	Girelli, R., Prejanò, S., Cataldo, I., Corbo, V., Martini, L., Scarpa, A., and Claudio, B. (2015). Feasibility and safety of electrochemotherapy (ECT) in the pancreas: a pre-clinical investigation. Radiology and oncology, 49(2), 147-154.	500
2	Mittal, L., Raman, V., Camarillo, I. G., Garner, A. L., Fairbanks, A. J., Dunn, G. A., and Sundararajan, R. (2017). Synergy of micro and nanosecond electrical pulses with chemotherapeutics on human cancer cell viability. In IEEE Conference on Electrical Insulation and Dielectric Phenomenon (CEIDP), 596-599.	500

Table S4. References for Figure S6.

Ref No.	Reference	Concentration (vol%)
1	Shen, Y., Xie, C., and Xiao, X. (2022). Black phosphorus-incorporated titanium dioxide nanotube arrays for near-infrared–triggered drug delivery. Journal of Drug Delivery Science and Technology, 72, 103400.	30

Table S5. References for Figure S7.

Ref No.	Reference	Pulse width (μs)
1	Skeate, J. G., Da Silva, D. M., Chavez-Juan, E., Anand, S., Nuccitelli, R., & Kast, W. M. (2018). Nano-Pulse Stimulation induces immunogenic cell death in human papillomavirus-transformed tumors and initiates an adaptive immune response. PLOS One, 13(1), e0191311.	0.1
2	Nuccitelli, R., McDaniel, A., Anand, S., Cha, J., Mallon, Z., Berridge, J. C., & Uecker, D. (2017). Nano-Pulse Stimulation is a physical modality that can trigger immunogenic tumor cell death. Journal for Immunotherapy of Cancer, 5(1), 1-13.	0.1

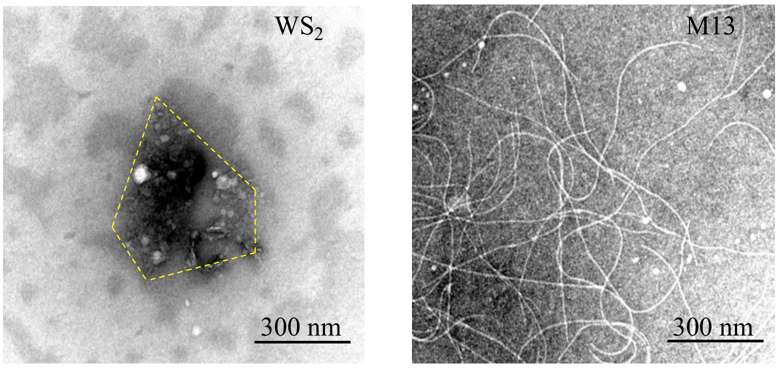

Figure S1. Transmission electron microscopy (TEM) images of the WS_2 nanosheet and M13 phage. The yellow dashed area contains the sheet-type structure.

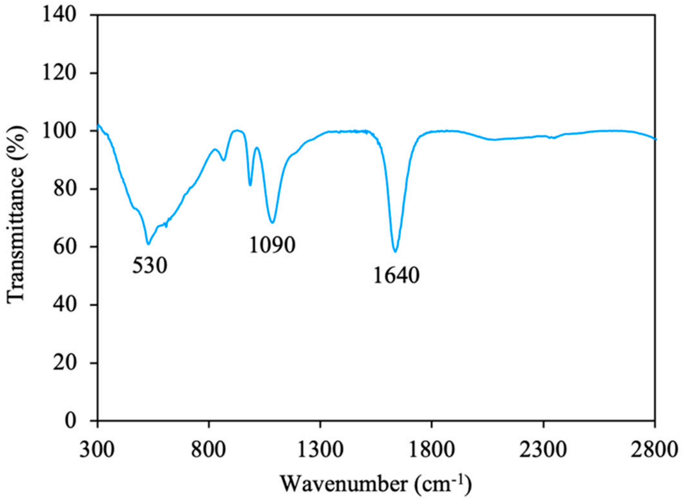

Figure S2. Fourier-transform infrared (FTIR) spectrum of the T nanostructure sample.

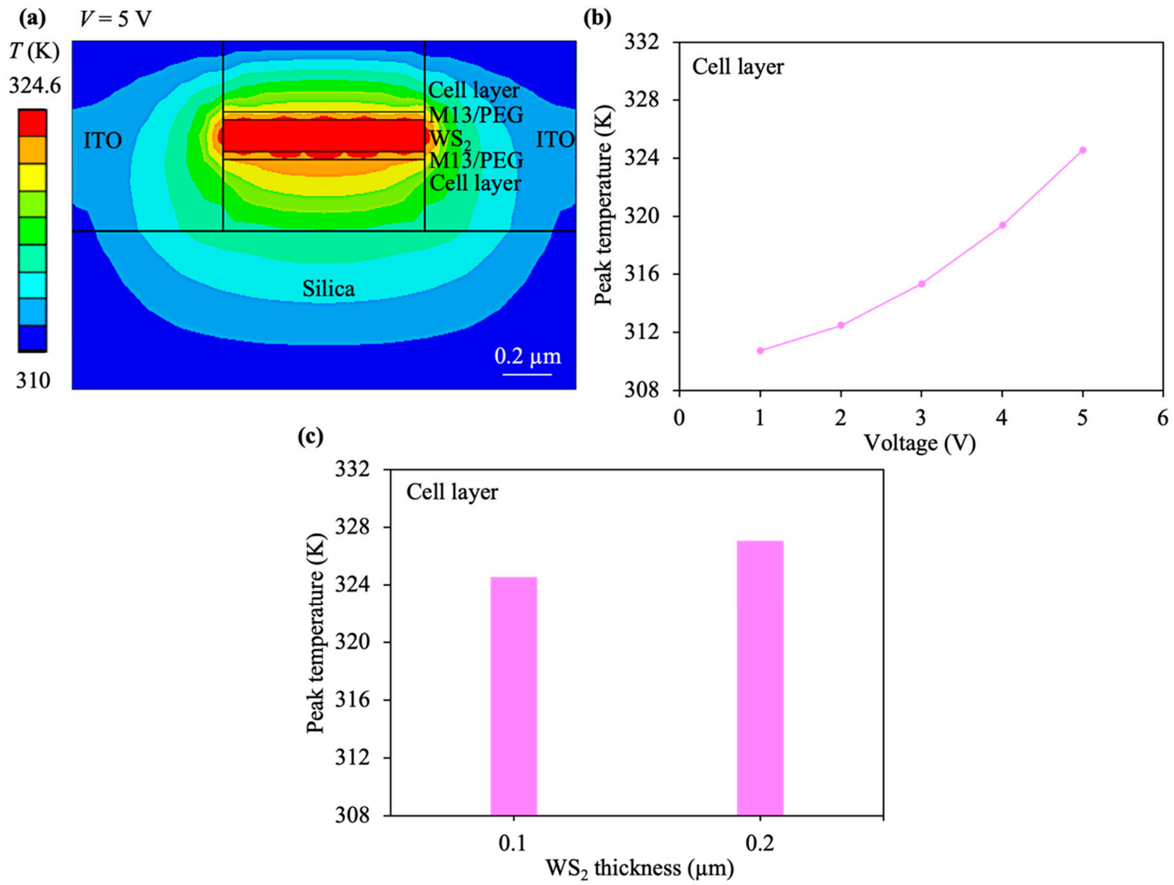

Figure S3. (a) Thermal-distribution analysis snapshot of the cell-layer/T-nanostructure model. The T nanostructure was inserted in the middle of the cell layer, and a square-based AC stimulus was applied. (b) Variation of the peak temperature in the cell layer observed for different stimulus amplitudes. (c) Variation of the peak temperature in the cell layer observed as a function of WS_2 thickness for 5 V input stimulus.

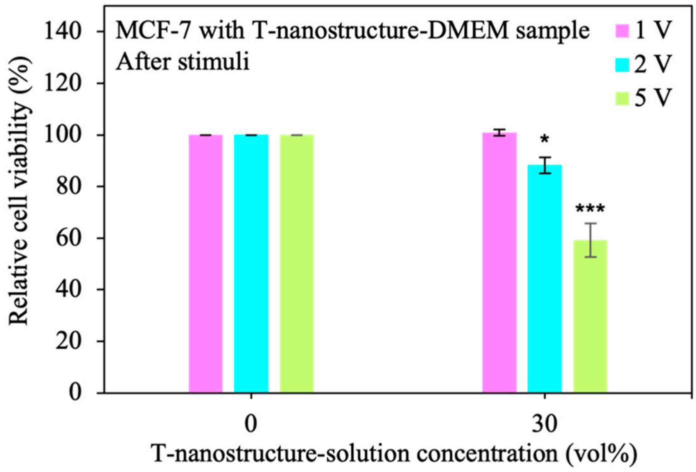

Figure S4. Relative viabilities of MCF-7 cells in T nanostructure-DMEM samples for different stimulus amplitudes. The error bars indicate the standard error of the mean (SEM) from three independent experiments ($n = 6$). The significance values were calculated using the Student's t-test and are indicated as follows: * ($p < 0.05$) and *** ($p < 0.001$).

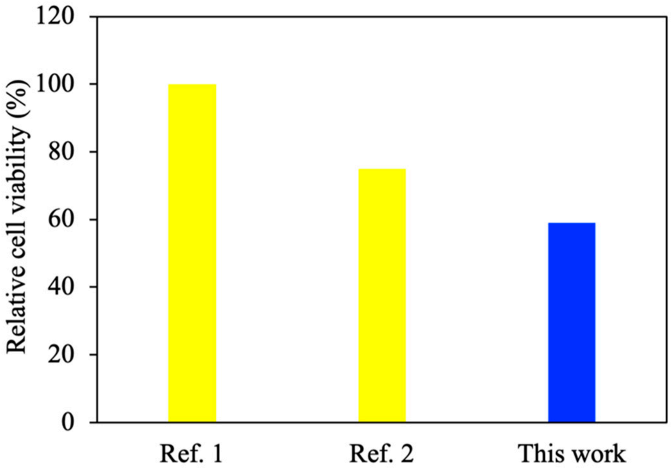

Figure S5. Comparison of the relative cell viability between the T-TT platform and existing electrothermal-based therapeutic systems. The information of the references can be found in Table S3.

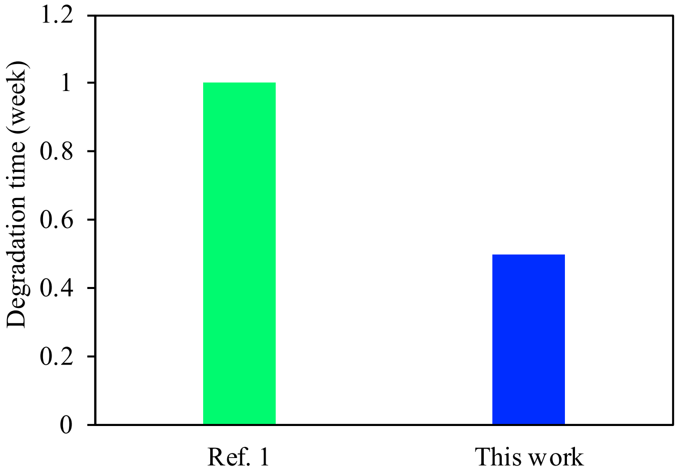

Figure S6. Comparison of the degradation time of the T nanostructure with state-of-the-art thermal agents in a physiological media. The information of the references can be found in Table S4.

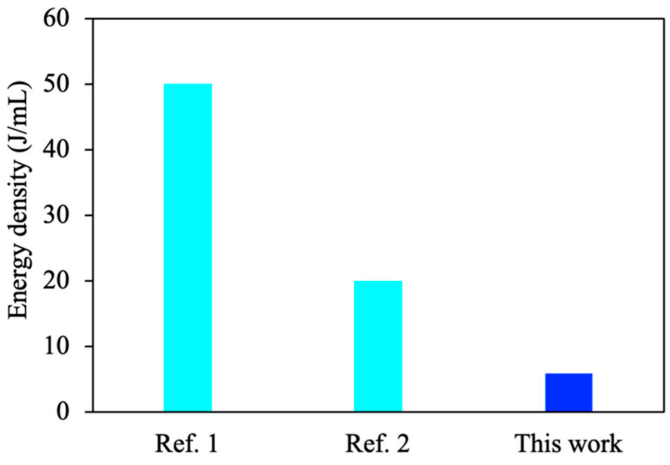

Figure S7. Comparison of the energy density between the T-TT platform and existing electrothermal-based therapeutic systems. The information of the references can be found in Table S5.
